# The Inverse Relationship between 25-Hydroxyvitamin D and Cancer Survival: Discussion of Causation

**DOI:** 10.3390/cancers5041439

**Published:** 2013-11-05

**Authors:** Trude E. Robsahm, Gary G. Schwartz, Steinar Tretli

**Affiliations:** 1 The Cancer Registry of Norway, Institute of Population-based Cancer Research, Oslo 0304, Norway; 2 Departments of Cancer Biology, Urology, and Epidemiology and Prevention, Wake Forest University Baptist Medical Center, Winston-Salem, NC 27157, USA

**Keywords:** vitamin D, cancer survival, causality, reverse causality, temporality

## Abstract

Cancer mortality rates vary inversely with geographic latitude and solar ultraviolet-B doses. This relationship may be due to an inhibitory role of vitamin D on cancer development. The relationship between vitamin D and cancer appears to be stronger for studies of cancer mortality than incidence. Because cancer mortality reflects both cancer incidence and survival, the difference may be due to effects of vitamin D on cancer survival. Here we review analytic epidemiologic studies investigating the relation between vitamin D, measured by circulating levels of 25-hydroxyvitamin D (25-OHD), and cancer survival. A relationship between low 25-OHD levels and poor survival is shown by most of the reviewed studies. This relationship is likely to be causal when viewed in light of most criteria for assessing causality (temporality, strength, exposure-response, biological plausibility and consistency). A serum level of 25-OHD around 50 nmol/L appears to be a threshold level. Conversely, there are several mechanisms whereby cancer could lower serum levels of 25-OHD. The severity of disease at the time of diagnosis and time of serum sampling are key factors to clarify the temporal aspect of these relationships. Evidence that vitamin D supplementation could retard the disease process or prolong survival time would be key evidence, but is difficult to generate. However, recent clinical trial results in prostate cancer support a role for vitamin D in this regard.

## 1. Introduction

An inverse association between ultraviolet-B radiation (UVB) and cancer mortality was suggested as early as 1941 [1]. UVB is the major source of vitamin D (the other source is dietary consumption, either from foods or supplements). Vitamin D synthesis begins when UVB (290–315 nm) converts pre-vitamin D in the skin into vitamin D (cholecalciferol). Vitamin D is then converted in the liver to the major circulating vitamin D metabolite, 25-Hydroxyvitamin D (25-OHD, calcidiol). Circulating levels of 25-OHD are known to vary by both geographic latitude (low at northern latitudes) and by season [2]. 

In 1980 Garland and Garland hypothesized that sunlight or vitamin D status accounted for the geographical differences in mortality from colon cancer, and that poor vitamin D status results in higher mortality [3]. In 1990, Schwartz and Hulka hypothesized that vitamin D deficiency increased the risk for clinical prostate cancer [4]. Throughout the 1990s and early 2000s, growing interest in vitamin D led to numerous ecological (correlational) studies on cancer incidence and mortality, most of which used indices of exposure to UVR as a proxy for vitamin D levels. Results from these studies confirmed inverse relationships between residential UVB doses and cancer mortality [5,6]. Results from studies on cancer survival, which used season as a proxy for vitamin D [7,8,9,10], supported the inverse gradient observed between UVB and cancer mortality in latitude studies. 

These ecologic studies prompted studies of cancer incidence and mortality at the individual level. From early 2000, numerous studies have investigated the association between circulating 25-OHD levels in individuals and incidence or mortality/survival for different cancers [11]. The results from these studies are equivocal. With respect to colorectal cancer, most studies support an inverse relationship between 25-OHD levels and risk [12,13,14,15]. Conversely, for prostate and breast cancer the relationship is less clear [13]. The Cohort Consortium Vitamin D Pooling Project of Rare Cancers (VDPP), reported results for the incidence of seven types of cancer, but no relationship between 25-OHD and risk was shown, with the exception of a possible positive association for pancreatic cancer [16]. Nor has a clear association with levels of 25-OHD been observed in mortality studies, except for the inverse association for colorectal cancer [17]. However, studies from the USA [18] and China [19] suggest a stronger relationship between UVB exposure and cancer mortality than for incidence. 

Death from cancer (cancer mortality) reflects both cancer incidence and survival (case fatality). Thus, at least part of the discrepancy in results for incidence and mortality studies may be due to an effect of 25-OHD on survival. Here we review studies that have investigated the relationship between serum vitamin D and cancer survival. Our aim is to discuss whether these associations reflect a causal effect of serum vitamin D on cancer survival, or whether they are attributable to the effect of cancer on circulating vitamin D levels (reverse causality). 

## 2. Review of Survival Studies

**Figure 1:**
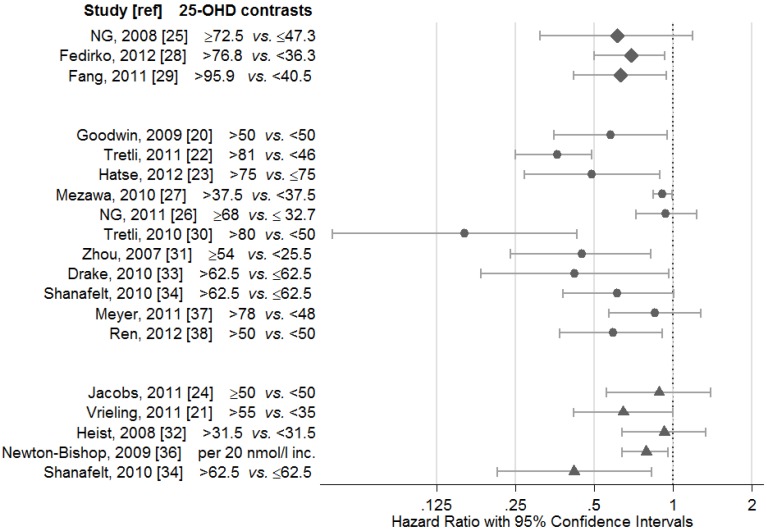
Hazard ratios with 95% confidence intervals from the reviewed studies, grouped by time of serum sampling, with regard to the time of diagnosis and start of treatment (25-OHD levels are given in nmol/L).

We searched PubMed for studies, published in English, that investigated the relationship between individual circulating levels of 25-OHD and cancer survival, that were published from January 2007 to May 2013. The reference lists of these studies also were used to identify studies. We identified 19 eligible studies that concerned circulating vitamin D levels and cancer survival in cancer patients. Fourteen of these (74%) reported an association between lower 25-OHD levels and inferior cancer survival (Table 1). No study reported an association between higher 25-OHD and poorer survival. Figure 1 gives the risk estimates with 95% Confidence Intervals (CI) from the studies. The risk estimates from comparison between groups with the largest contrast were chosen. For studies that tabulated risk estimates according to the lowest versus highest exposure category, the inverse of the values are presented.

### 2.1. Breast Cancer

Six studies were available for breast cancer. Goodwin *et al*. reported results from a prospective study of women with early stage breast cancer [20]. 25-OHD was assayed in plasma samples collected recently after diagnosis (mean 58 days), prior to treatment. An almost two-fold risk of distant recurrence was observed in women with 25-OHD levels <50 nmol/L. The hazard ratio (HR) for death was 1.73 (95% CI 1.05–2.86), when compared to patients with levels >72 nmol/L. A large German study of postmenopausal breast cancer cases only, reported an improved overall survival (HR 1.55, 95% CI 1.00–2.39) and distant-free survival (HR 2.09, 95% CI 1.29–3.41) in patients with serum 25-OHD >55 nmol/L, compared with levels <35 nmol/L [21]. Serum samples were drawn a median of 83 days after diagnosis. The significant association with overall survival was restricted to cases with samples collected before the start of chemotherapy. As a part of a Norwegian cohort study reported by Tretli *et al.* using serum drawn within 90 days of diagnosis, pre- or postmenopausal breast cancer patients were followed up for cancer-specific death [22]. When women in the highest quartile of 25-OHD (≥86 nmol/L) were compared with those in the lowest quartile (<50 nmol/L), the risk of death was significantly reduced (HR 0.42, 95% CI 0.21–0.82). Similar results were observed in a large cohort study by Hatse and colleagues, including non-metastatic breast cancer patients [23]. Serum samples were drawn at diagnosis, prior to any treatment. Serum levels of 25-OHD >75 nmol/L were associated with improved survival. Low 25-OHD levels (<50 nmol/L) were also associated with larger tumor size at diagnosis, however, the results were significant for postmenopausal patients only. Conversely, a nested case-control study within the Women’s Healthy Eating and Living Study (WHEL) reported no significant associations between 25-OHD levels and breast cancer recurrence or death when comparing patients with 25-OHD levels ≥50 and <50 nmol/L [24]. Importantly, this study was based on serum that, on average, was obtained 2 years post-diagnosis.

### 2.2. Colorectal Cancer

Based on the Nurses’ Health Study and the Health Professionals Follow-Up study, Ng *et al*. investigated the association between vitamin D and survival in patients with colorectal cancer [25]. Plasma levels of 25-OHD were assessed at a median of 72 months prior to diagnosis. The results suggest a (non-significantly) lower risk of cancer death (HR 0.61, 95% CI 0.31–1.19) in patients within the highest quartile of 25-OHD (>72.5 nmol/L) compared with the lowest quartile (<47.3 nmol/L). In a subsequent study by Ng and colleagues, no difference in survival was seen between patients with 25-OHD levels >68 and ≤32.7 nmol/L [26]. This study was based on whole-blood samples drawn after diagnosis of late stage disease, but prior to chemotherapy. Statistical power was considerably limited in this study as only 18% of the patients had 25-OHD levels >75 nmol/L. Results from a Japanese cohort study, based on serum samples drawn during surgery and prior to any other treatment, showed a better prognosis for patients with 25-OHD levels >37.5 nmol/L, compared to lower levels (HR 0.91, 95% CI 0.84–0.99) [27]. Also the Norwegian study of Tretli *et al*. suggests inferior survival in patients with low 25-OHD levels [22], but the study only included 52 colon cancer cases. The HR for death from colon cancer was 0.20 (95% CI 0.04–1.10) for patients with 25-OHD levels in the highest quartile (≥77 nmol/L) compared with those in the lowest quartile (<44 nmol/L). Recently, results from a cohort study within the European Prospective Investigation into Cancer and Nutrition (EPIC) reported similar results [28]. An improved survival was seen in patients with 25-OHD levels >76.8 nmol/L (HR of 0.69, 95% CI 0.50–0.93) compared to patients with levels <36.3 nmol/L.

### 2.3. Prostate Cancer

Low serum levels of 25-OHD may also influence survival from prostate cancer. A study based on the Health Professionals Follow-up Study (HPFS) and the Physicians Health Study (PHS) showed that patients with 25-OHD levels <40.5 nmol/L were more likely to die from prostate cancer (HR 1.59, 95% CI 1.06–2.39) compared with levels >95.9 nmol/L [29]. From both cohorts, prediagnostic serum samples were used. The association was largely explained by the association between low 25-OHD levels and cancer of advanced stage and higher Gleason score. The association tended to be stronger when restricting the analyses to patients with samples collected within five years of the cancer diagnosis. Similar results were observed in a Norwegian study of prostate cancer patients, based on serum samples collected ±3 months from the date of the cancer diagnosis [30]. The risk of cancer death in patients with 25-OHD levels >80 nmol/L was 0.16 (95% CI 0.05–0.43) relative to patients with levels <50 nmol/L. A risk reduction was also seen in patients with 25-OHD levels 50–79 nmol/L (RR 0.33, 95% CI 0.14–0.77). 

### 2.4. Lung Cancer

Two studies investigated the association between 25-OHD and survival in patients with non-small cell lung cancer. An association between high 25-OHD levels and improved survival was suggested for patients with early-stage disease, in particular for subgroups [31], but no clear association was seen for advanced stage patients [32]. The first study was based on serum samples drawn at diagnosis and prior to treatment, whereas the latter used serum drawn within 2 months of diagnosis and after start of treatment. The Norwegian cohort study, based on pre-treatment serum samples from 210 lung cancer patients, all histological types, demonstrated inferior survival in patients with low serum 25-OHD [22]. The hazard ratio for cancer death in patients in the highest quartile of 25-OHD (≥76 nmol/L) was 0.18 (95% CI 0.11–0.29) compared with the lowest quartile (<41 nmol/L).

### 2.5. Hematologic Cancers

Lower vitamin D levels (25-OHD levels <62.5 nmol/L) were associated with inferior survival in non-Hodgkin’s lymphoma patients, both with B-cell (HR 1.99, 95% CI: 1.27–3.13) and T-cell lymphoma (HR 2.38, 95% CI: 1.04–5.41) [33]. The 25-OHD assessment was based on serum samples drawn within 120 days after diagnosis, prior treatment for 66% of the patients and during or after treatment for 34%. The same group [34] studied patients with chronic lymphocytic leukaemia (CLL), including two cohorts: the *discovery* (390 newly diagnosed CLL patients) and a *confirmation cohort* (153 previously untreated CLL patients). Improved survival was reported for patients with 25-OHD levels >62.5 nmol/L. After a median follow-up of 3 years, the discovery cohort showed a higher risk of death for patients with 25-OHD levels <62.5 nmol/L (HR 2.39, 95% CI 1.21–4.70) compared with higher levels. After a median follow-up of 9.9 years of the confirmation cohort, the HR for overall survival was 1.63 (95% CI 0.99–2.69) when comparing patients with 25-OHD ≥62.5 nmol/L with lower levels. A similar association was observed in the Norwegian study, including 145 lymphoma patients with no further specification [22]. The HR for lymphoma death was 0.39 (95% CI 0.18–0.83) when comparing 25-OHD levels ≥77 nmol/L with levels <44 nmol/L. In a study by Pardanani *et al.*, including patients with myeloproliferative disease and myelodysplastic syndromes, no association was seen between 25-OHD insufficiency (levels <50 nmol/L) and overall survival [35].

### 2.6. Other Cancers

Results from a study on melanoma patients, based on serum samples drawn within 6 months after diagnosis, showed a hazard ratio for relapse-free survival of 0.79 (95% CI 0.64–0.96) per 20 nmol/L increase in serum 25-OHD, and accordingly 0.83 (95% CI 0.68–1.02) for overall survival [36]. For head and neck cancer, no association was observed between 25-OHD levels and cancer outcomes, either for recurrence (HR 1.12, 95% CI 0.65–1.93), second primary cancer (HR 0.72, 95% CI 0.40–1.30) or death (HR 0.85, 95% CI 0.57–1.28), comparing highest (>78 nmol/L) and lowest quartiles (<48 nmol/L) [37]. A Chinese study that examined the relation between pre-treatment 25-OHD levels and overall survival in gastric cancer patients reported a HR of cancer death in patients with 25-OHD levels >50 nmol/L of 0.59 (95% CI 0.37–0.91) compared with levels <50 nmol/L [38].

**Table 1:** The studies investigating the relationship between circulating level of 25-OHD and cancer survival, grouped by time for serum sampling with regard to time of diagnosis and treatment.

Study [ref]	Cancer ^1^	Size (n)	Follow-up, years ^4^	Adjustments ^5^	Relation 25-OHD/survival	Main findings
***Studies based on serum collected prior to diagnosis***						
NG, 2008 [25]	CRC	304	0–14 ^c^	1,2,5,6,11,12,15–17	(+)	Non-significant reduced risk of cancer death for levels ≥72.5 nmol/L (HR 0.61,95% CI 0.31–1.19) compare with levels ≤47.3 nmol/L
Fedirko, 2012 [28]	CRC	1202	73 ^a^ months	1,2,5,6,9,11,12, 15–17,20	+	Reduced risk of cancer death in patients with levels >76.8 nmol/L (HR 0.69, 95% CI 0.50–0.93) compared with levels <36.3 nmol/L
Fang, 2011 [29]	PC	185	10 ^a^	1,2,6,9,11	(+)	Increased risk of cancer death if 25-OHD <40.5 nmol/L (HR 1.59, 95% CI 1.06–2.39) compared with levels >95.9 nmol/L (not significant when adjust for Gleason score/stage)
*Studies based on serum collected at or recently after diagnosis and prior to treatment*						
Goodwin, 2009 [20]	BC ^2^	512	11.6 ^a^	1–5	+	Increased risk of cancer death (HR 1.73, 95% CI 1.05–2.86) in patients with 25-OHD levels <50 compared with levels >50 nmol/L
Tretli, 2011 [22]				1,2,12,15	+	Reduced risk of cancer death if high 25-OHD levels
	BC	251	9.3 ^a^			BC: HR 0.42 (95% CI 0.21-0.82), ≥86 *versus* <50 nmol/l
	CC	52	7.3 ^a^			CC: HR 0.20 (95% CI 0.04-1.10), ≥77 l *versus* <44 nmol/L
	LC	210	1.6 ^a^			LC: HR 0.18 (95% CI 0.11-0.29), ≥76 *versus* <41 nmol/L
	NHL/HL	145	6.3 ^a^			NHL/HL: HR 0.39 (95% CI 0.18-0.83), ≥77 *versus* <44 nmol/L
Hatse, 2012 [23]	BC ^2^	1800	0–9^c^	1–3,5,6,12,15	+	Reduced risk of cancer death when 25-OHD levels >75 nmol/L (HR 0.49, 95% CI 0.27-0.89) compared with levels ≤75 nmol/L (significant for postmenopausal BC only)
Mezawa, 2010 [27]	CRC	257	0–5 ^c^	1–3,12,15,17	+	Reduced risk of death if 25-OHD levels >37.5 nmol/L (HR 0.91, 95% CI 0.84-0.99) compared with levels <37.5 nmol/L
NG, 2011 [26]	CRC ^3^	515		1,2,6,12,15,18,19	0	No association between 25-OHD levels and survival. RR was 0.94 (95% CI 0.72–1.23) for 25-OHD levels ≥68 compared with levels ≤32.7 nmol/L (82% were <75 nmol/L)
Tretli, 2010 [30]	PC	160	3.7 ^b^	1,5,12,18	+	Reduced risk of cancer death in patients with 25-OHD >80 nmol/L (HR 0.16, 95% CI 0.05–0.43) or 50–80 nmol/L (HR 0.33, 95% CI 0.14–0.77) compared with levels <50 nmol/L
Zhou, 2007 [31]	NSLC ^2^	447	72 ^b^ months	1,2,9,12,15,19	(+)	All: No association between 25-OHD and survival. IB-IIB stage patients: reduced risk of death if 25-OHD levels ≥54 nmol/L (HR 0.45, 95% CI 0.24–0.82) compared with levels <25.5 nmol/L
Drake, 2010 [33]	NHL	983	34.8 ^b^ months	1–3,15,18,20	+	Increased risk of death for B-cell (HR 1.99, 95% CI 1.27–3.13) and T-cell (HR 2.38, 95% CI 1.04–5.41) lymphoma types, if 25-OHD levels ≤62.5 nmol/L (Serum drawn prior to (n = 649), during or after (n = 334) treatment)
Shanafelt, 2010 [34]	CLL	153	9.9 ^b^	1–3,15	(+)	The *confirmation cohort*: non-significant increased risk of cancer death if 25-OHD levels ≤62.5 nmol/L (HR 1.63, 95% CI 0.99–2.69)
Meyer, 2011 [37]	HNC	522	4.4 ^b^	1,2,6,9,10,12,16,19,21	0	No association between 25-OHD level and risk of death. HR for highest (>78 nmol/L) versus lowest quartile (<48 nmol/L) was 0.85 (95% CI 0.57–1.28)
Ren, 2012 [38]	GC	197	0–8.3 ^c^	1,2,5,6,9,12,15,16,19,21	+	Reduced risk of cancer death in patients with 25-OHD levels >50 nmol/L (HR 0.59, 95% CI 0.37–0.91) compared with levels <50 nmol/L
***Studies based on serum collected after diagnosis and after start of treatment***						
Jacobs, 2011 [24]	BC ^2^	512	7.3 ^a^	1,2,4–8,19,22	0	No association with risk of cancer death (OR 1.13, 95% CI 0.72–1.79), comparing 25-OHD levels <50 and ≥50 nmol/L
Vrieling, 2011 [21]	BC	1295	5.8 ^b^	1–5,12–14	+	Increased risk of cancer death if 25-OHD levels <35 nmol/L (HR 1.55, 95% CI 1.00–2.39) compared with levels >55 (postmenopausal BC only)
Heist, 2008 [32]	NSLC ^3^	294	42 ^b^ months	2,15,18	0	No associations between 25-OHD and survival, comparing 25-OHD levels <31.5 nmol/L with higher levels (HR 1.08, 95% CI 0.75–1.57)
Newton-Bishop, 2009 [36]	CMM	872	4.7 ^b^	1,2,6,12,15,16	+	Improved relapse-free survival per 20 nmol/L increase in serum 25-OHD (HR 0.79, 95% CI 0.64–0.96)
Shanafelt, 2010 [34]	CLL	390	3 ^b^	1–3,15	+	The *discovery cohort*: Increased risk of cancer death if 25-OHD levels ≤62.5 nmol/L (HR 2.39, 95% CI 1.21–4.70)
Pardanani, 2011 [35]	MPN	409	0–300 ^c^ months	1,2,15,16,18	0	No association between 25-OHD levels an survival, comparing patient with 25-OHD levels ≥62.5 and <62.5 nmol/L. Estimates were not possible to abstract (information on pre or post treatment sampling is not given)

^1^ Cancer disease abbreviations: BC: breast cancer; CRC: colorectal cancer; CC: colon cancer; PC: prostate cancer; NSLC: non-small cell lung cancer; LC: lung cancer; NHL: non-Hodgkin’s lymphoma; CLL: chronic lymphocytic leukemia; HL: Hodgkin’s lymphoma; MPN: myeloproliferative neoplasm’s; CMM: cutaneous malignant melanoma; GC: gastric cancer; HNC: head & neck cancer; ^2^ early stage disease, ^3^ advanced stage disease, ^4^ time of follow-up; ^a^ mean, ^b^ median or ^c^ range; ^5^ Adjustment variables: 1 = age; 2 = cancer stage; 3 = nodal stage; 4 = estrogen receptor; 5 = tumor grade/differentiation; 6 = BMI; 7 = ethnicity; 8 = calcium intake; 9 = smoking; 10 = vitamin D intake; 11 = physical activity; 12 = season of blood collection; 13 = tumor size; 14 = diabetes; 15 = sex; 16 = tumor site; 17 = time/period; 18 = baseline performance status; 19 = treatment; 20 = area of residence; 21 = alcohol consumption; 22 = education.

## 3. Discussion

The studies reviewed above indicate a relationship between low vitamin D levels (expressed by 25-OHD levels) and poor survival in cancer. The key question is: *How do we interpret this relationship*? Specifically, do low levels of 25-OHD contribute to poorer survival in cancer patients, or do patients with poorer survival have lower 25-OHD levels because of their cancer? Criteria usually involved in making a causal determination for epidemiological associations include: the temporal relationship between the variables, strength of the association, exposure-response relationship, biological plausibility, consistency, reversibility, and coherence [39]. These aspects are discussed in turn below. Paramount to this discussion is an evaluation of temporality. 

### 3.1. Temporality

This causal criterion means that low levels of vitamin D should precede the disease process. This criterion is difficult to satisfy completely. Because many cancers, e.g., prostate cancer, have latent periods of 20 years or more, we rarely know when the disease process starts, and time spent in the process may differ considerably between individuals. The presence of cancer is first realized at the time of diagnosis, which is the start of follow-up in a survival analysis. There are several mechanisms whereby cancer could affect serum levels of 25-OHD. These include the effects of cancer on vitamin D metabolism, nutritional absorption of vitamin D and effects of reduced sunlight exposure. Lack of appetite, fatigue and/or weight loss are common symptoms of cancer and often reflect the severity of the disease. It is reasonable to believe that such symptoms may influence a patients’ condition and behavior and hence, their circulating level of 25-OHD. 

There are also endocrine mechanisms whereby the presence of cancer can influence the 25-OHD levels. These may be different in different cancers. For example, in some cancers, e.g., breast and ovarian cancer, serum calcium levels may rise as a paraneoplastic (remote) effect of the tumor. This will result in an inhibition of 1-alpha hydroxylase in the kidney [40,41,42]. Consequently, less 25-OHD is converted to 1,25-dihydroxyvitamin D (1,25(OH)_2_D). The net result is that this may cause a slight increase in serum 25-OHD, since less 25-OHD is converted to 1,25(OH)_2_D. Lytic bone disease, as may occur in advanced breast cancer, is a frequent cause of hypercalcemia. The pathophysiology is the reverse in advanced prostate cancer where blastic metastases cause serum calcium levels to fall [43]. This will cause more 25-OHD to be converted to 1,25(OH)_2_D as part of the feedback-loop to correct hypocalcemia, and will lower the circulation pool of 25-OHD. 

The best information that we have on disease severity is stage at the time of diagnosis. Most studies included in this review have taken disease severity into account in the analyses, e.g., using stage or other known prognostic factors (Table 1). Generally, these adjustments have had little effect on the relationship between 25-OHD level and cancer survival. For example, Tretli *et al.* [22] confirmed that the relationship was seen even when the analyses were stratified by stage of disease. 

The time of collection of serum for 25-OHD analyses is important to this discussion. If the disease process and symptoms lower the 25-OHD levels and cause the association, we might not expect to observe the association in studies based on serum samples drawn years prior to the diagnosis. These samples should be less contaminated by the disease process. Three of the studies reviewed here [25,28,29] were based on serum drawn a long time prior to diagnosis, but the results still suggest an inverse relationship between 25-OHD levels and cancer death (Figure 1). In the study by Fang *et al.* [29], the association tended to be stronger for those with the most recent blood draw (within 5 years of the diagnosis), which might, in part, reflects reverse causation. 

Moreover, some cancer therapies, including chemotherapy, are likely to have depressant effects on vitamin D [44], and, thus, potentially confound the relationship between vitamin D and survival. Four of the reviewed studies were based exclusively on blood samples collected after the start of treatment [21,24,32,36]. Two of these studies showed a significant inverse relationship between 25-OHD levels and cancer survival [21,36]. It is noteworthy that the study by Jacobs *et al*. [24] was based on serum drawn a long time after diagnosis, and only early stage breast cancer cases with a fairly good prognosis were included. The study by Heist *et al*. [32] included advanced lung cancer patients, all of whom have a poor prognosis. Further, the 25-OHD levels in this patient group were quite low, which may permit only a weak contrast between the patients. For a small proportion of the prostate cancer patients in the study by Tretli *et al*. [30], serum was drawn after hormonal treatment. The inverse relationship between 25-OHD and cancer death, however, was stronger for this patient group than for patients with serum drawn prior to treatment. Also in the study by Shanafeldt *et al*. [34], a stronger association was observed for CLL patients given treatment prior to blood draw (Figure 1). In the study by Vierling *et al*. [21], however, the association between 25-OHD and survival was restricted to cases with serum drawn prior to treatment. Based on these results, it is difficult to determine whether the treatment itself or an interaction between treatment and vitamin D influence the 25-OHD level and cause the relationship. Eleven studies, however, were based on samples drawn at the time of diagnosis or recently after (Figure 1), and most of these suggest an association between lower 25-OHD levels and inferior cancer survival. In eight of these, serum was drawn prior any systemic treatment [20,22,23,27,31,34,37,38] and the relationship observed could not result from influence of treatment. Thus, we conclude that treatment *per se* does not explain the association observed between 25-OHD and survival.

### 3.2. Strength and Exposure-Response Relationship

The magnitude of the association is an important criterion for assessing causality. We note that the association between low 25-OHD level and poor cancer survival is relatively strong. There is not convincing evidence for a typical dose-response relationship, however. Rather, the data better approximate a threshold model whereby low levels of 25-OHD are associated with an increased risk of death, but then plateau. It is important to recognize that a hypothesis that vitamin D insufficiency is associated with reduced survival does not imply that vitamin D suprasufficiency (levels above the threshold) is associated with even better survival. In studies where vitamin D levels are categorized in quartiles, the analyses indicate that the main gap in survival rate is at about 50 nmol/L [22,25,28,30,33,34,38]. It is noteworthy that in analyses of such studies, one must be aware of the existence of a threshold level. When assuming a linear exposure-response relationship in the analysis, it is possible to end up with a diluted and misleading result.

### 3.3. Biological Plausibility

The biological plausibility for a causal relation between low levels of circulating 25-OHD and inferior survival is unambiguously supported by laboratory and experimental studies. The receptor for the hormonal form of vitamin D (1,25(OH)_2_D) has been demonstrated in almost all tissues, including neoplastic cells [45]. Additionally, analogs of the vitamin D hormone are potent inhibitors of angiogenesis, cell growth and metastasis. Anti-tumor effects of vitamin D in animal models for cancers are well-documented [45]. Detailed discussions about vitamin D actions in cancers of the breast [46], prostate [47], colorectum [48], hematological malignancies [49] and other cancers [50], have established the plausibility of a causal role between vitamin D and cancer. However, as emphasized by Jacobs *et al.* [51], it has been challenging to reconcile the results from laboratory studies, demonstrating the biological mechanisms of the active hormone form of vitamin D (1,25(OH)_2_D), and the observational studies, using levels of circulating 25-OHD. The vitamin D metabolite 25-OHD has been considered to be far less potent than 1,25(OH)_2_D, as it binds the receptor with approximately 1/500–1/1000th the affinity of 1,25(OH)_2_D. Nevertheless, autocrine non-renal synthesis of 1,25(OH)_2_D from 25-OHD (via the enzyme 1-OHase) occurs in many tissues, including the prostate [52], colon and breast [53]. Unlike the kidney, where the hydroxylation of 25-OHD is tightly regulated, the hydroxylation of 25-OHD in non-renal tissues appears to be substrate-dependent. Hence, the synthesis of 1,25(OH)_2_D in many tissues likely depends on the serum level of 25-OHD [54,55]. 

Thus, the discrepancy between the laboratory data, which largely involve the vitamin D hormone and its analogs, and epidemiology, which largely involves 25-OHD, may not be so difficult to bridge. There is a growing body of data, both *in vitro* and *in vivo*, which support a role for many vitamin D metabolites, including 25-OHD and even vitamin D itself, as an inhibitor of cancer growth. For example, Barreto and colleagues showed that 25-OHD at physiological doses inhibited the proliferation of primary cultures of prostate cancer cells that possess 1-OHase [56]. Similar data were shown by Chen *et al*. [57]. More recently, reasoning that cholecalciferol (vitamin D_3_) would be converted endogenously to 25-OHD, Swami *et al.* demonstrated that dietary vitamin D exhibited equivalent anticancer effects as 1,25-dihydroxyvitamin D in mouse xenograft models of prostate and breast cancer [58]. These data provide compelling biological evidence for a potential role for 25-OHD as a mediator of cancer survival. 

### 3.4. Consistency

The consistent findings of an inverse relationship between 25-OHD levels and risk of cancer death in three of four of the reviewed studies points towards a causal relation. Although a few of the reviewed studies report no association, it is noteworthy that none of the studies report a positive relation between vitamin D levels and risk of cancer death. Further, the inverse relationship is suggested for many different cancers, which may indicate that vitamin D influences general mechanisms in cancer biology. In addition, the inverse relationship is seen in studies that were carried out in countries with different cultures and different health care systems.

### 3.5. Reversibility

In the absence of data from randomized clinical trials it is impossible to evaluate whether vitamin D can retard or reverse the pathophysiology of cancer in humans. Such studies face both ethical and practical problems. First, there is no consensus about an optimum serum level for vitamin D in cancer patients. This level is important to define since high levels of 25-OHD have been implicated to increase all-cause mortality [59]. Second, the multiple sources of vitamin D represent practical challenges in the administration of such a study, as it may be difficult to control the influence of vitamin D from UVR exposure, dietary intake and supplement use. Third, knowledge in the population about possible beneficial effects of vitamin D may contaminate the intervention. Despite these difficulties, there are clinical trial data that support a potential role for vitamin D. In an innovative study, Wagner *et al.* conducted a double-blind randomized trial of vitamin D in doses ranging from 400–40,000 International Units (IU), administered to men scheduled for prostatectomy for cure of their prostate cancer [60]. High doses of vitamin D (40,000 IU) were demonstrated to result in higher levels of 1,25(OH)_2_D in the prostate, confirming in vivo the autocrine synthesis of 1,25(OH)_2_D by human prostatic cells demonstrated by Schwartz *et al*. in 1998 [52]. Moreover, serum Prostate Specific Antigen (PSA), a marker for prostatic growth, was significantly lower in men given high dose vitamin D [60].

### 3.6. Coherence

This criterion describes how different types of data, e.g., from cell culture and geography, support or fail to support the same hypothesis. It is noteworthy that results from the studies examining the relation between 25-OHD levels and survival are consistent with studies based on latitude and UVR exposure. As it is difficult to imagine that geographical variation in persons’ behavior or different treatment procedures could cause such a relationship in the latitude-based ecological studies, we conclude that the coherence criterion is largely satisfied.

## 4. Conclusions

Results from the survival studies show a consistent relationship between low 25-OHD levels and inferior cancer survival. The association between low serum 25-OHD and survival is seen in different populations, among different cancer sites, and among different stages of disease, and persists after adjustment for suspected confounders. The association seems not to be linear and a serum level of 25-OHD ~50 nmol/L may be a threshold level. Although the consequences of cancer and its treatment also may cause a decline in serum vitamin D levels, judgment of key criteria for causality (temporality, strength, exposure-response, biological plausibility and consistency) support a causal relationship between vitamin D and cancer survival. Evidence that vitamin D supplementation could retard the disease process or prolong survival time in humans would be key evidence. Although ethical constraints make randomized trials difficult to conduct, emerging evidence from a clinical trial in prostate cancer support this concept.
